# Improving case duration accuracy of orthopedic surgery using bidirectional encoder representations from Transformers (BERT) on Radiology Reports

**DOI:** 10.1007/s10877-023-01070-w

**Published:** 2023-09-11

**Authors:** William Zhong, Phil Y. Yao, Sri Harsha Boppana, Fernanda V. Pacheco, Brenton S. Alexander, Sierra Simpson, Rodney A. Gabriel

**Affiliations:** 1 Division of Perioperative Informatics, Department of Anesthesiology, University of California, La Jolla, San Diego, CA USA; 2 School of Medicine, University of California, La Jolla, San Diego, CA USA; 3 Department of Biomedical Informatics, University of California San Diego Health, La Jolla, San Diego, CA USA

**Keywords:** Natural language processing, Machine learning, Operating room, Orthopedic surgery

## Abstract

**Purpose:**

A major source of inefficiency in the operating room is the mismatch between scheduled versus actual surgical time. The purpose of this study was to demonstrate a proof-of-concept study for predicting case duration by applying natural language processing (NLP) and machine learning that interpret radiology reports for patients undergoing radius fracture repair.

**Methods:**

Logistic regression, random forest, and feedforward neural networks were tested without NLP and with bag-of-words. Another NLP method tested used feedforward neural networks and Bidirectional Encoder Representations from Transformers specifically pre-trained on clinical notes (ClinicalBERT). A total of 201 cases were included. The data were split into 70% training and 30% test sets. The average root mean squared error (RMSE) were calculated (and 95% confidence interval [CI]) from 10-fold cross-validation on the training set. The models were then tested on the test set to determine proportion of times surgical cases would have scheduled accurately if ClinicalBERT was implemented versus historic averages.

**Results:**

The average RMSE was lowest using feedforward neural networks using outputs from ClinicalBERT (25.6 min, 95% CI: 21.5–29.7), which was significantly (P < 0.001) lower than the baseline model (39.3 min, 95% CI: 30.9–47.7). Using the feedforward neural network and ClinicalBERT on the test set, the percentage of accurately predicted cases, which was defined by the actual surgical duration within 15% of the predicted surgical duration, increased from 26.8 to 58.9% (P < 0.001).

**Conclusion:**

This proof-of-concept study demonstrated the successful application of NLP and machine leaning to extract features from unstructured clinical data resulting in improved prediction accuracy for surgical case duration.

**Supplementary Information:**

The online version contains supplementary material available at 10.1007/s10877-023-01070-w.

## Introduction

The operating room (OR) is a substantial source of both revenue and overhead for a medical system [1], [2]. Inefficient utilization of OR time significantly increases overhead costs [3] [4]. Common efficiency metrics include first case delays (first surgical procedure in the operating room started on time), turnover times (time between each surgical procedure), and scheduled case duration accuracies (i.e., the difference between scheduled surgical case duration and the actual case duration) [5]. Maximizing the efficiency for each OR time point is important to minimize the incidence of under-utilization (unused operating room time in a set block of time) and over-utilization (operating room time used over the set block of time) of the operating room. Furthermore, accurate prediction of surgical case duration has been shown to have the highest association with operating room efficiency in an outpatient surgery center [5]. Cases that routinely take longer than expected result in OR overutilization, increasing costs in the form of overtime pay and higher employee dissatisfaction and burnout rates. OR underutilization from shorter than anticipated case times leads to increased staff idle time, which is connected to up to a 60% higher cost [6].

The first step in the surgical scheduling process is to create a list of cases and estimate durations. A method to determine case duration times is directly from the surgeon, who individually reserves a block of OR time based on surgeon’s expectations and experience. However, this strategy has its limitations, as surgeons may overestimate up to 32% of the time and underestimate 42% of the time [7]. Another method is using the historic average, which can be determined using the electronic health record (EHR) and previous data for a particular procedure and/or surgeon. Surgical time using EHR-generated historic averages for case duration have been shown to have a marginally higher accuracy when compared to direct surgeon estimation [8]. Limitations of depending on historic averages include lack of patient and procedural-specific factors taken into account for overall case duration. Furthermore, it may not account for pathology-specific factors that can also impact case duration.

Much of the data for patient-specific and procedure-specific data is encoded as structured clinical data and can be easily extracted and evaluated, but pathology-specific data is often encoded in unstructured clinical free text. Natural language processing (NLP) and machine learning may potentially be used process unstructured free text to generate a model for case duration. Open reduction and internal fixation (ORIF) of radial fractures are a common orthopedic surgery performed in the outpatient setting [9–11]. Surgical time for radius fracture repair has been shown to be influenced by the severity and the fracture pattern, which are communicated in the radiology report of the wrist X-ray [9]. This data would be useful in predicting case duration but is usually encoded as unstructured clinical text. The objective of this study was to demonstrate proof-of-concept for using NLP and machine learning to interpret unstructured clinical data and use that to predict surgical case duration. We hypothesized that surgical times predicted by NLP and machine learning interpretation of the radiology read for patients undergoing radius fracture surgery will be more accurate than surgical times obtained from the historic averages approach.

## Materials and methods

### Study population

This was a retrospective study involving secondary use of de-identified patient data and the consent requirement was waived by University of California San Diego’s Institutional Review Board (Human Research Protections Program, protocol #801,106). The study analyzed all patients that underwent open reduction internal fixation (ORIF) of radius fractures from 2020 performed at a single outpatient surgery center affiliated with the institution. Patients who did not have preoperative X-ray images available of the wrist were excluded from the analysis.

### Primary outcome and data collection

The primary outcome measurement was surgical case duration in minutes. Data collected included radiology results of the preoperative wrist X-ray, which comprised of a document with unstructured text describing the findings of the wrist fracture per the radiologist. Further data collected included patient age, sex, weight, American Society of Anesthesiologists Physical Status (ASA PS), surgeon performing the procedure, and the scheduled surgical case duration. The scheduled surgical case duration was calculated at the institution based on the historic average of actual case duration of the last three procedures performed by that surgeon. There were no missing data points.

### Statistical analysis

Python (v3.10.4) was used for all statistical analysis. All code is provided as Supplementary Files and have been uploaded to github (https://github.com/UCSDGabrielLab/BERT/blob/main/bert_case_1.ipynb.

https://github.com/UCSDGabrielLab/BERT/blob/main/case_models_nlp.ipynb). In summary, various machine learning models were developed with or without using NLP-processed radiology reports. These models incorporated all other features as inputs (age, sex, surgeon, weight, ASA PS classification score, scheduled case duration, scheduled skin minutes, and surgeon) to predict actual surgical case duration. This was then compared to the predictions from the historic averages. First, the dataset was divided into training and test sets with a 70:30 split using a randomized splitter—the “train_test_split” method from the sci-kit learn library. Then, using the training set (70% of data), 10-fold cross-validation was performed to calculate the average root-mean-square error (RMSE), measured in minutes, and the mean-squared error (MSE). For both metrics, the 95% confidence intervals (CI) were provided. We then used the best performing model to predict surgical times on the test set (30% of data) and quantified the proportion of times surgical cases would have been scheduled accurately (within 15% of the scheduled case duration) if a predictive model was implemented versus using historic averages.

### Natural language processing models

Three different machine learning classification models without the use of NLP-processed radiology reports were initially evaluated: linear regression, random forest classifier, and feedforward neural network. The same three models were also evaluated using an NLP algorithm on the radiology reports - bag-of-words (BOW). The final predictive model used Bidirectional Encoder Representations from Transformers (BERT) combined with feedforward neural networks.

#### Bag-of-words

A document-term matrix was created where each column represented a unique word from the input space and each row contained the raw counts for each word. For this approach, all non-ASCII characters and non-word characters were removed, words were lemmatized to their roots, and words were converted to lowercase. The outputs from each note were then fed into the subsequent machine learning model for prediction. For the BOW approach, all features and the document-term matrix were used as input. There were no hyperparameters adjusted for the BOW approach.

#### Bidirectional encoder representations from transformers

**Fig. 1:**
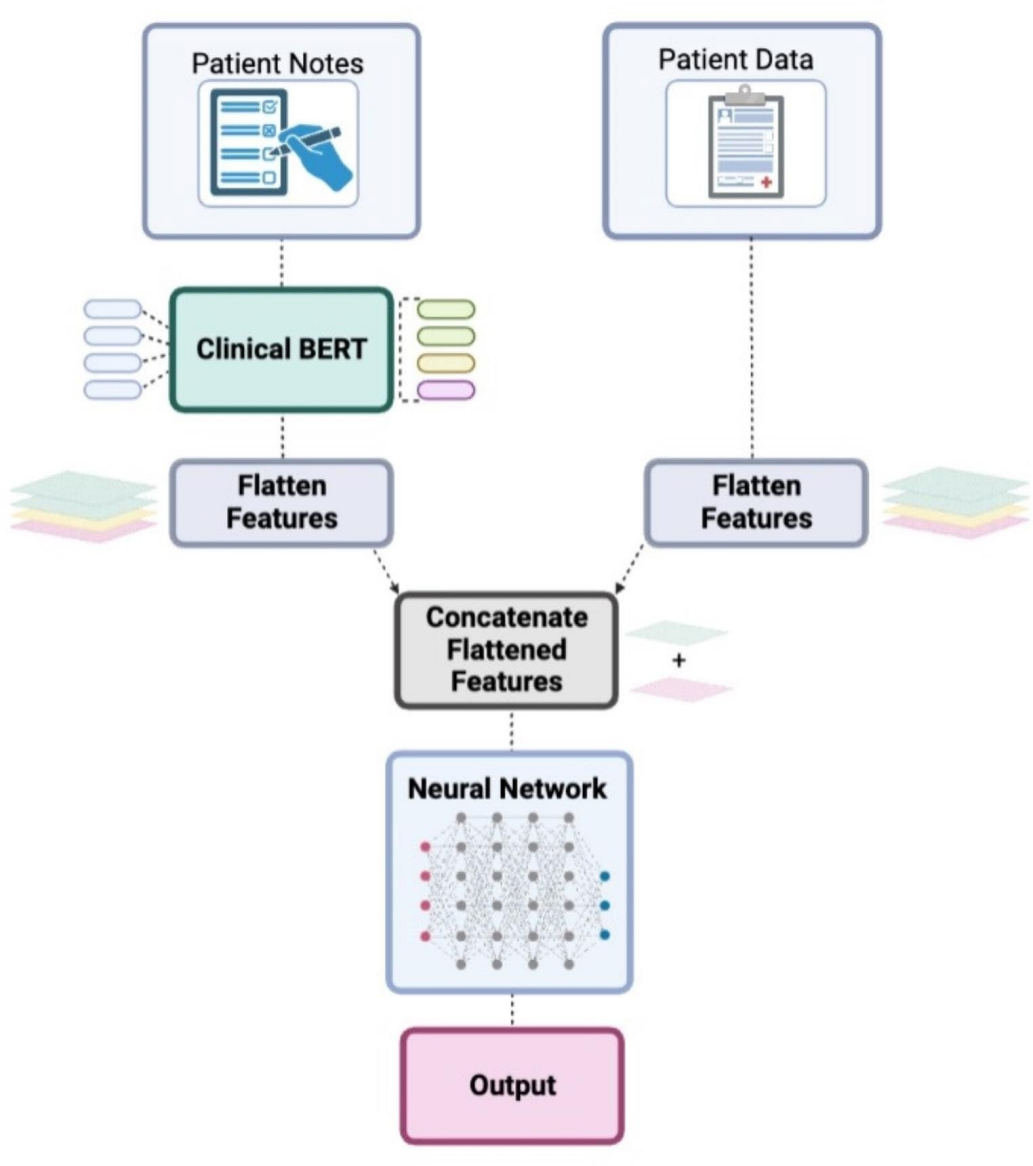
Illustration of methodology of implementation of Bidirectional Encoder Representations from Transformers (BERT) on processing unstructured clinical text data for the machine learning models. Radiology reports (unstructured clinical text documents) were processed via BERT in which its output was combined with patient static data. This combined output was then inputted into a subsequent feedforward neural network to predict surgical case duration

ClinicalBERT, a modified version of Bidirectional Encoder Representations from Transformers (BERT) pretrained on patient clinical notes, was implemented (Fig. 1). For this approach, only non-ASCII and non-word characters were removed from all notes from the study population (training and test set). Due to the BERT limited to 512 tokens as input, patient notes were truncated to the first 512 tokens. The ClinicalBERT approach used all features including the preprocessed patient notes. For each machine learning model, hyperparameter tuning based on k-folds cross validation on the training set was performed. The best model was then evaluated on the test set and compared with the baseline.

### Machine learning models

We performed various machine learning models to predict actual case duration. All models were compared to the baseline model. Below, we describe the hyperparameters tuned for each model-type and the final values assigned to each.

#### Baseline model

A baseline model was constructed using the historic averages (scheduled case duration). This was based on the average case duration of the last three times the surgeon performed this surgery. This model served as a reference for comparison with the other models.

#### Linear regression

Linear regression models model multiple inputs to a continuous outcome (i.e. case duration). A newton-cg solver regression model was used and the optimal value for *C* (the strength of the regularization is inversely proportional to *C*) was found to be 1.0 for both the non-NLP and BOW approaches.

#### Random forest regressor

The random forest is an ensemble approach (a method which takes the predictions of multiple machine learning algorithms and combines them together to make a more accurate prediction) of decision trees. Alone, decision trees have proven to be effective in a variety of problems. To tune the hyperparameters, repeated k-folds cross-validation was performed to find the optimal number of trees to be 50 and 150 for the non-NLP and BOW approaches via manual hyperparameter tuning.

#### Feedforward neural network

Using the Keras interface for the TensorFlow library, a feedforward neural network for the non-NLP, BOW, and ClinicalBERT approaches was built. The activation function used was the rectified linear unit function. The final output layer used the linear activation function, and the overall model used the Adam optimizer. To tune the hyperparameters, repeated k-folds cross-validation was performed to find the optimal parameter values for number of hidden layers, number of neurons per hidden layer, epochs, batch size, and learning rate. These were found to be 1, 256, 200, 16, and 0.01, respectively, for the non-NLP approach and 1, 128, 100, 64, and 0.01, respectively, for the BOW approach. Two input layers containing the non-text features and ClinicalBERT embeddings were directly fed into a feedforward neural network. The pretrained ClinicalBERT’s weights were frozen. The final hyperparameters included 2 hidden layers, which contained 256 and 128 neurons, 10 epochs, a batch size of 8, and a learning rate of 0.00005 with the “decay” parameter set to 0.00001 to be optimal. All feedforward neural networks were found to be most optimal when using the mean absolute error loss function.

## Results

### Study population

A total of 201 patients who underwent ORIF of radial fractures were included in this study, in which the median [quartiles] surgical case duration was 85 min [65, 90 min]. The majority of the patients were ASA PS 2 (50.2%) and were men (74%). The median [quartile] age was 55 years [34, 63 years] and the median patient weight was 75.8 kg [64.8, 86.1 kg] (Table 1).

**Table 1:** Baseline characteristics of study population

Features	n	%
Total	201	
ASA PS Classification Score		
1	68	33.8
2	101	50.2
3	32	15.9
Age (years), median [quartile]	55 [34, 63]	
Weight (kg), median [quartile]	75.8 [64.8, 86.1]	
Men (versus women)	74	36.8
Scheduled Skin Time, median [quartile]	85 [65, 90]	
Surgeon		
other	22	10.9
A	83	41.3
B	96	47.8

Abbreviations: ASA PS, American Society of Anesthesiologists

### Natural language processing and machine learning models

**Fig. 2:**
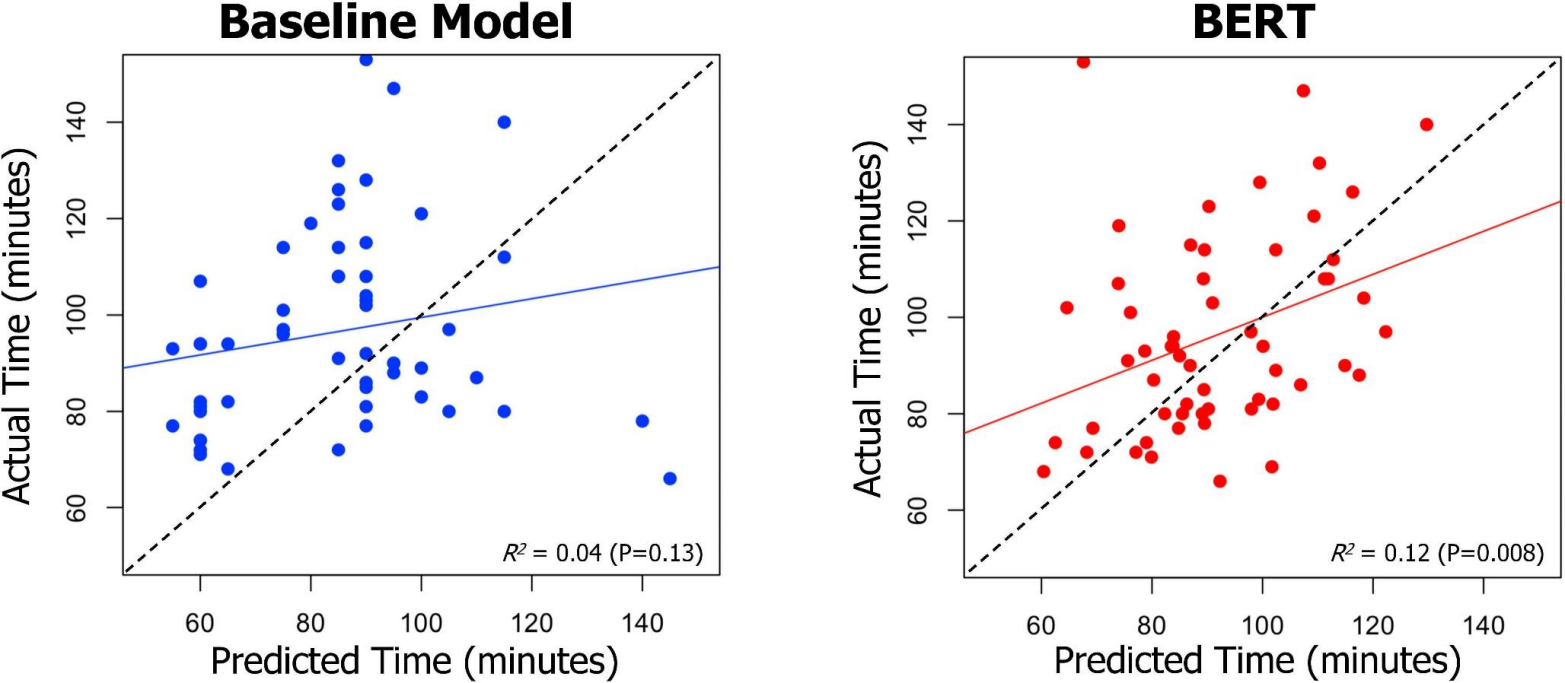
Plots illustrating the correlation between actual surgical versus predicted time in the baseline (historic averages of surgical procedure) versus BERT model. Abbreviations: BERT, Bidirectional Encoder Representations from Transformers

We next assessed the predictive ability of various machine learning models for surgical case duration and compared those to a baseline model – which calculated scheduled case duration based on the historic average of that case (the average the last three times that particular surgeon performed this operation). For each model, the average RMSE was calculated via 10-fold cross-validation using the training set. The RMSE for the baseline historic averages model was 39.3 (95% CI 30.9, 47.7) minutes. First, we implemented a logistic regression, feedforward neural network, and random forest model that inputted non-radiology report features (e.g. surgeon, sex, ASA PS classification score, weight, and age) to predict surgical time. Logistic regression, feedforward neural networks, and random forest had an RMSE of 43.3 (95% CI 35.1, 51.4), 36.6 (95% CI 31.1, 42.2), and 37.9 (95% CI 33.9, 41.9) minutes, respectively. Next, we processed the radiology reports using BOW which produce an output used in the machine learning models. The logistic regression, feedforward neural networks, and random forest models’ RMSE improved to 39.3 (95% CI 31.7, 46.9), 34.5 (95% CI 28.4, 40.6), and 35.2 (95% CI 29.6, 40.8) minutes, respectively. However, when ClinicalBERT output was applied to a feedforward neural network model, the RMSE dropped significantly to 25.6 (95% CI 21.5, 29.7) minutes and had statistically significantly better performance compared to the historic average model (P < 0.001) (Table 2). The *R*^*2*^ of BERT versus historic averages model in predicting case duration was 0.12 versus 0.04, respectively (Fig. 2).

**Table 2:** Results of the various models predicting actual case duration compared to the baseline model. Neural networks were feedforward neural networks

Models	Mean Squared Error (95% Confidence Interval) [minutes]	Root Mean Squared Error (95% Confidence Interval) [minutes]
baseline model	1670.9 (993.4, 2348.4)	39.3 (30.9, 47.7)
logistic regression	1987.9 (1243.3, 2732.4)	43.3 (35.1, 51.4)
neural network	1394.4 (989.6, 1799.2)	36.6 (31.1, 42.1)
random forest	1464.9 (1169.6, 1760.2)	37.9 (33.9, 41.9)
logistic regression (bag of words)	1645.7 (1047.9, 2243.6)	39.3 (31.7, 46.9)
neural network (bag of words)	1255.5 (843.4, 1667.6)	34.5 (28.4, 40.6)
random forest (bag of words)	1293.5 (886.6, 1700.3)	35.2 (29.6, 40.8)
neural network using features from ClinicalBERT	685.5 (471.6, 899.5)	25.6 (21.5, 29.7)

Abbreviations: BERT, bidirectional encoder representations from transformers

We next calculated the proportion of times surgical cases would have scheduled accurately if ClinicalBERT was implemented versus historic averages. Accurate scheduling was defined as actual case duration falling within 15% of the scheduled duration. When applied to the test set, the ClinicalBERT model and historic averages model accurately predicted 33/56 (58.9%) and 15/56 (26.8%) accurately, respectively (P < 0.001).

## Discussion

In this study, we demonstrated that predictions for surgical case duration were improved when NLP and machine learning were used to extract data from the radiology read of the preoperative wrist X-ray. Specifically, ClinicalBERT showed statistically significant improvement in predicting surgical case duration compared to the historic averages approach. We hypothesized that there would be salient features within the radiology report which would describe the complexity and severity of the injury, which would then translate to increasing duration of surgery. From our results, we demonstrated that processing these radiology reports provided important information for better predicting surgical case duration compared to when historic averages are used to calculate surgery time. These historic averages were based on previous times that surgeon performed this surgery, but did not take into account the variability in fracture disease and anatomy.

Previous research has estimated the cost to the hospital for every unused OR minute to range from $22 and $133 [12]. Therefore, by properly utilizing the OR time, operationalizing case duration prediction using this approach could lead to a significant decrease in expenses. This estimate is indicative of a direct savings in OR overhead costs and likely underestimates the cumulative downstream impact across the hospital system, which includes optimizing hospital bed utilization and reducing hospital length of stays. Therefore, seemingly small savings in unused OR time can compound to substantial cost reductions across a healthcare system. For example, the ClinicalBERT model reduced the RMSE by approximately 15 min. If this translates into 15 min per case of unused time, that could approximately translate to $300 to $2,000 saved per case. Likely multiple cases are performed in an OR per day, thus the savings could be even higher. Although, there are a plethora of other factors unaccounted for in this calculation and thus it is difficult to determine exact savings without a well-designed prospective study.

Numerous earlier studies have examined strategies for surgical scheduling optimization. Many studies have demonstrated the superiority of using historic averages to predict the duration of a surgical case over that of individual surgeons [8], [9]. Other studies have looked at the use of mathematical modeling to enhance case sequencing [13], [14] or to predict case duration in the absence of historical data [15]. A few studies have described the use of machine learning to improve predictions for surgical case duration of various surgeries [16–21]. These studies demonstrated a benefit of applying machine learning to EHR data to improve accuracy of surgical case duration, but the features were extracted from only structured data points.

Unstructured clinical data, such as radiology reports, are encoded as narrative text, which grants the clinician freedom to capture both subjective and objective perspectives within the document. Because of the absence of structure, it may be more challenging to consistently extract features from unstructured data via an automated approach without the use of NLP [22]. In this study, features containing information on the severity and type of radius fracture were extracted from the official radiology interpretation of the wrist X-ray using NLP and machine learning. Of the tested NLP models, ClinicalBERT performed the best in predicting case duration and was significantly more accurate compared the traditional approach. This suggests that the information found within the radiology reports contain vital information that impact surgical case duration – data that would not otherwise be put into account consistently without NLP algorithms.

Accurate predictions of surgical case duration help to optimize the surgery schedule [21]. This study demonstrated that utilization of NLP within a machine learning framework improved accuracy of predicting case duration for ORIF for radial fractures at a single ambulatory surgery center. This proposed model could be applied to other surgical procedures with high variability in duration of surgery that are dependent on imaging reports to identify severity and fracture pattern. Future studies could apply this concept to large volumes of surgical procedures and subsequently evaluate the cost-savings to healthcare systems from optimizing the surgery schedule.

### Limitations

There are several limitations to the current study design - mainly attributable to the retrospective nature of the data. With retrospective studies, there are potential biases that could be related to inaccuracies of data points, which could skew results. However, there was no evidence of obvious errors in the data. For example, there were no instances of extreme and, thus, obviously inaccurate values for the recorded case durations (e.g. unlikely values for surgical duration) or features (e.g. impossible recorded age or weight). If there were systemic errors, it would introduce overfitting issues as the models would train on inaccurate data entries. Another weakness is external validity due to the relatively small sample size from a single institution. Furthermore, there is concern for generalizability of this model to other datasets as only data from a single ambulatory surgery center was utilized. Another limitation is the lack of transparency regarding the impact of textual features processed by ClinicalBERT on the predictions. For example, it is unclear if the pathology-specific features within the radiology reports were the important features for prediction. In addition, there are several other potentially important features not included in the predictive model that may be relevant, including the current surgical volume in the operating rooms, time of day, time of the year, and staffing shortages. The results of this study should be interpreted as a proof-of-concept study that may drive larger scale studies. Furthermore, the results of this study do not provide causal explanations, but rather reports associations of various features to the outcome of interest. Ideally, a large prospective study using data from multiple institutions and operating room settings (outpatient and inpatient) should be performed.

## Conclusion

We demonstrated that improvements to machine learning models predicting surgical case duration could be made when using NLP (ClinicalBERT) to process radiology imaging reports. These reports provide more context on the severity and fracture pattern, which may influence surgical case duration. Such models may aid operating room managers in creating the daily surgery schedule to minimize unused OR time and avoid over-utilization from inaccurate historical averages data or surgeon estimation. The reduction of mismatch between scheduled surgical time and actual surgical time can lead to improved patient care and downstream saving to hospital systems.

### Electronic supplementary material

Below is the link to the electronic supplementary material.

Supplementary Material 1

Supplementary Material 2
